# A Dynamic Multimodality Imaging Assessment of Right Ventricular Thrombosis in a Middle-Aged Man with Lymphocytic Interstitial Pneumonia: The Additive Role of Tissue Doppler Imaging

**DOI:** 10.3390/jcm14062035

**Published:** 2025-03-17

**Authors:** Andrea Sonaglioni, Alessandro Lucidi, Francesca Luisi, Antonella Caminati, Gian Luigi Nicolosi, Gaetana Anna Rispoli, Maurizio Zompatori, Michele Lombardo, Sergio Harari

**Affiliations:** 1 Division of Cardiology, IRCCS MultiMedica, 20123 Milan, Italy; michele.lombardo@multimedica.it; 2 Division of Pneumology, Semi-Intensive Care Unit, IRCCS MultiMedica, 20123 Milan, Italy; alessandro.lucidi@unimi.it (A.L.); francesca.luisi@multimedica.it (F.L.); antonella.caminati@multimedica.it (A.C.); sergio.harari@unimi.it (S.H.); 3 Division of Cardiology, Policlinico San Giorgio, 33170 Pordenone, Italy; gianluigi.nicolosi@gmail.com; 4 Division of Radiology, IRCCS MultiMedica, 20123 Milan, Italy; gaetanaanna.rispoli@multimedica.it; 5 DIMES Department, University of Bologna, 40126 Bologna, Italy; maurizio.zompatori@unibo.it; 6 Department of Clinical Sciences and Community Health, Università di Milano, 20122 Milan, Italy

**Keywords:** right ventricular thrombosis, pulmonary embolism, multimodality imaging assessment, tissue Doppler imaging, prognosis

## Abstract

**Background:** Right ventricular thrombosis (RVT) is rarely detected in clinical practice. Depending on its aetiology, RVT may originate from a deep venous thrombosis (type A) or in situ (type B). Type A is characterized by increased mobility and frequent pulmonary embolization, whereas type B is nonmobile and is associated with significant right ventricular (RV) dilatation and dysfunction. **Methods:** A type B RVT complicated by subsegmental pulmonary embolism (PE) was diagnosed in a 46-year-old man with acute-on-chronic respiratory failure secondary to acute exacerbation of interstitial lung disease. He underwent a multimodality imaging assessment of the RV mass that comprehensively incorporated TTE, TEE, contrast-enhanced chest CT, and LGE-CMR. **Results:** During the clinical course, a serial echocardiographic assessment of the RV mass allowed for a dynamic evaluation of its features and cardiac haemodynamics. Conventional TTE was implemented with colour tissue Doppler imaging (TDI) and pulsed wave (PW) TDI to improve the visualization of the RV mass and to objectively measure its mobility. The increased RVT mass peak antegrade velocity (>10 cm/s) was predictive of subsequent RVT fragmentation and PE. **Conclusions:** Colour TDI and PW-TDI may aid in the differential diagnosis of RV masses and may improve the prognostic risk stratification of patients with right-sided intracardiac masses.

## 1. Introduction

Right ventricular thrombosis (RVT) is a rare and frequently underdiagnosed life-threatening condition [1]. It can be detected in 2.6–18% of patients with pulmonary embolism (PE) [2,3,4].

The main risk factors for RVT are the following: a younger age, previous bleeding events, congestive heart failure, intracardiac procedures, cancer, episodes of syncope, a transient systolic blood pressure <100 mmHg, and arterial oxyhaemoglobin saturation <90% [2]. Hypercoagulability, secondary to Factor 5 Leiden or antithrombin 3 mutations, is another factor that has been associated with RVT occurrence [5]. In certain cases, hypercoagulability may be secondary to occult malignancy [6]. Loeffler’s endocarditis [7] and takotsubo cardiomyopathy, affecting the right ventricular (RV) apex [8], are other causes of RVT, which are rarely detected in clinical practice.

Most patients with RVT have significant RV dilatation and dysfunction. Cases of RVT have also been reported in patients with acute inferior-wall myocardial infarction complicated by RV infarction [9]. From a pathophysiological point of view, mechanisms for RVT formation in situ are related to the so-called “Virchow’s triad”, which consists of blood stasis, endothelial dysfunction, and concomitant hypercoagulability.

Based on its aetiology, RVT is classified in types A, B, and C. Type A is a highly mobile serpiginous thrombus, which is entrapped within the Chiari’s network and/or in right heart cavities, that originates from the embolization of a deep venous thrombosis (DVT) and is commonly associated with PE. Type B is nonmobile, originates in situ, and is associated with cardiac abnormalities. Type C has intermediate characteristics between type A and B [10,11].

RVT is generally asymptomatic before the occurrence of complications such as PE or paradoxical stroke [12]. However, the mortality rate associated with RVT is high, ranging between 27% and 100% [1,2], especially if it is complicated by PE. Indeed, the prognosis of these patients is primarily related to the haemodynamic consequences of RVT rather than its characteristics.

Due to the severity of RVT occurrence and complications, immediate diagnosis and rapid prognostic risk stratification of RVT patients are mandatory.

Two-dimensional transthoracic echocardiography (TTE) and transoesophageal echocardiography (TEE) are the first-line imaging modalities for detecting and monitoring RVT [11]. It is noteworthy that both TTE and TEE have a higher sensitivity and specificity for detecting left ventricular thrombosis rather than RVT. The first assessment of RVT by TTE may be difficult in an emergency setting and in patients with tachy-arrhythmias, haemodynamic instability, or poor acoustic windows [13]. Additionally, approximately 50% of RV thrombi are identified by using off-axis echocardiographic sections [11]. Therefore, RVT presence may be considerably underestimated in clinical practice.

Given that the echogenicity of thrombotic formation may be indistinguishable from that of surrounding myocardium, TTE may provide limited information concerning the differential diagnosis of intracardiac masses [14]. In the setting of poor acoustic windows and/or suboptimal TTE imaging, contrast echocardiography may considerably improve the endocardial definition by enhancing the blood pool–myocardial interface, thus facilitating RVT detection and characterization [15].

Due to its unique capability in providing an accurate and reproducible assessment of RV structure, function, and tissue characterization, cardiac magnetic resonance (CMR) with late gadolinium enhancement (LGE) has shown higher sensitivity and specificity than TTE and/or TEE for detecting RVT [16,17].

Finally, contrast-enhanced chest computed tomography (CT) represents another useful technique for evaluating RVT, due to its rapid acquisition and high spatial and temporal resolution [18,19,20].

Herein, we present a challenging case of type B RVT that was diagnosed by a multimodality imaging approach in a 46-year-old man with acute-on-chronic respiratory failure secondary to acute exacerbation of interstitial lung disease.

## 2. Clinical Course

A 46-year-old man (BSA 1.92 m^2^; BMI 24.5 Kg/m^2^) without previous cardiovascular events, who was affected by anti-Mi2 dermatomyositis and lymphocytic interstitial pneumonia (LIP) with chronic respiratory failure and was treated with 50 mg of azathioprine daily, 25 mg prednisone daily, 700 mg of intravenous (IV) rituximab once weekly, and oxygen therapy (1 to 6 L per minute), was admitted to the Emergency Department (ED) of our institution due to ongoing dyspnoea, chest pain, and general malaise. At the hospital admission, the patient’s arterial oxygen saturation (SaO2) in ambient air was 66%, their blood pressure was 110/70 mmHg, their heart rate was 102 b.p.m., and their body temperature was 36.3 °C. A blood gas analysis showed hypoxemia (PaO2 = 39.6 mmHg) and hypocapnia (PaCO2 = 27.2 mmHg), pH = 7.3, and a lactate level of 7.7 mmol/L (normal range: 0.36–1.25 mmol/L). Blood tests revealed a serum haemoglobin amount of 12.8 g/dL; a serum white blood cell count of 13,900 × 10^6^/L (normal range: 4000–11,000 × 10^6^/L); a serum Neutrophil–Lymphocyte Ratio (NLR) of 18.6; a serum creatinine level of 1.57 mg/dL; a serum troponin I level of 0.42 ng/mL (normal range 0.00–0.04 ng/mL); a serum C-reactive protein (CRP) amount of 58 mg/L (normal range: 0–5 mg/L); a serum D-dimer of 4215 microg/L (normal range: 1–500 microg/L); and a serum N-terminal pro-B-type natriuretic peptide (NT-proBNP) level of 6474 pg/mL (normal range: <125 pg/mL).

**Figure 1:**
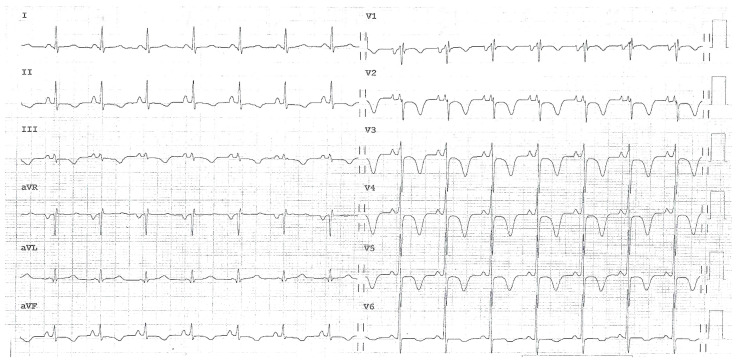
A 12-lead electrocardiogram showing a sinus rhythm with normal atrio-ventricular conduction, mild right ventricular conduction delay, and deep T-wave inversion in right precordial leads and inferior leads, suggesting right ventricular overload.

The ECG recorded in the ED showed a sinus rhythm with normal atrio-ventricular conduction, mild RV conduction delay, and deep T-wave inversion in right precordial leads and inferior leads, suggesting RV overload (Figure 1).

**Figure 2:**
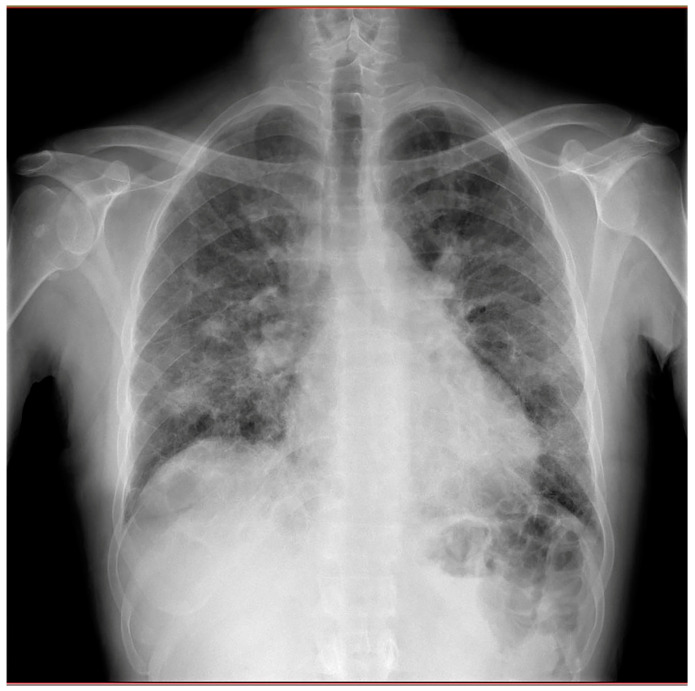
Chest X-rays revealing diffuse fibrosing interstitial lung disease with multiple bilateral parenchymal opacities, with no clear evidence of inflammatory foci.

Chest X-rays showed diffuse fibrosing interstitial lung disease with multiple bilateral parenchymal opacities, with no clear evidence of inflammatory foci (Figure 2).

**Figure 3:**
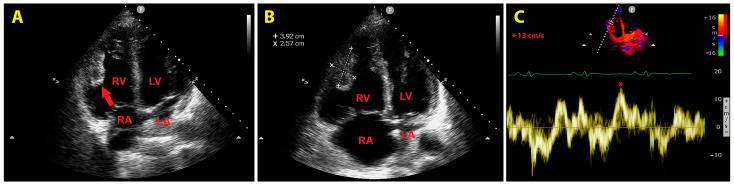
Transthoracic echocardiography. Right-ventricular-focused apical four-chamber view. (**A**) Large sessile echogenic formation with hyperechoic edges (red arrow), attached to the mid-apical portion of the right ventricular free wall and protruding into the RV cavity. (**B**) Measurement of the right ventricular mass size. (**C**) Assessment of the right ventricular mass peak antegrade velocity by pulsed wave tissue Doppler imaging. The right ventricular mass showed a pattern of incoherent motion, totally discordant and independent from the surrounding myocardial tissue. LA, left atrium; LV, left ventricle; RA, right atrium; RV, right ventricle. The symbol * indicates the right ventricular mass peak antegrade velocity.

An urgent bedside echocardiogram highlighted a significant dilatation of right-sided cardiac chambers (RV-to-left ventricular basal diameter ratio = 2.4; RV inflow tract diameter = 60 mm) and mild hypokinesia of the RV lateral wall, as assessed by tricuspid annular plane systolic excursion (TAPSE) magnitude (17 mm). A moderate tricuspid regurgitation was present. The peak tricuspid regurgitation velocity (TRV) was 3.4 m/s, indicating a high probability of pulmonary hypertension (PH). The inferior vena cava was significantly dilated (transverse diameter = 2.8 cm), with inspiratory excursions < 50%. Accordingly, the estimated systolic pulmonary artery pressure (sPAP) was 60 mmHg. From the RV-focused apical four-chamber view, a large sessile echogenic formation with hyperechoic edges (size: 3.9 cm × 2.6 cm), attached to the mid-apical portion of the RV free wall and protruding into the RV cavity, was detected (Figure 3A,B). By placing a 5 mm sample volume of pulsed wave (PW)-tissue Doppler imaging (TDI) at the level of the mobile portion of the RV mass, an RV mass peak antegrade velocity (Va) of 13 cm/s was obtained. Moreover, on PW-TDI, the RV mass showed a pattern of incoherent motion, totally discordant and independent from the surrounding myocardial tissue (Figure 3C).

Careful observation allowed for the detection of akinesia of the RV mid-apical wall. Therefore, McConnell’s sign was excluded. Compared to the right-sided cardiac chambers, the left-sided cavity chambers’ size was reduced. The left ventricle showed a D-shaped left ventricular configuration due to the flattening of the interventricular septum caused by the significant RV overload. The left ventricular ejection fraction (assessed by a modified Simpson’s biplane method) was preserved (estimated value = 60%). A first degree of diastolic dysfunction (E/A ratio < 1 on transmitral PW Doppler) was diagnosed in the presence of normal left ventricular filling pressure, as measured by the E/average e’ ratio (estimated value = 5.2). The mitral and aortic valves were normal, whereas a mid-systolic notch of the RV outflow tract PW-Doppler envelope, compatible with PH, was detected. The urgent TTE was compared with a previous one, performed electively three months earlier, which showed normal biventricular cavity sizes, normal biventricular systolic function, and normal sPAP (estimated value: 27 mmHg). Accordingly, the actual TTE findings were considered to be suggestive of acute RV overload due to PH, complicated by the RV mass of nonunivocal interpretation.

**Figure 4:**
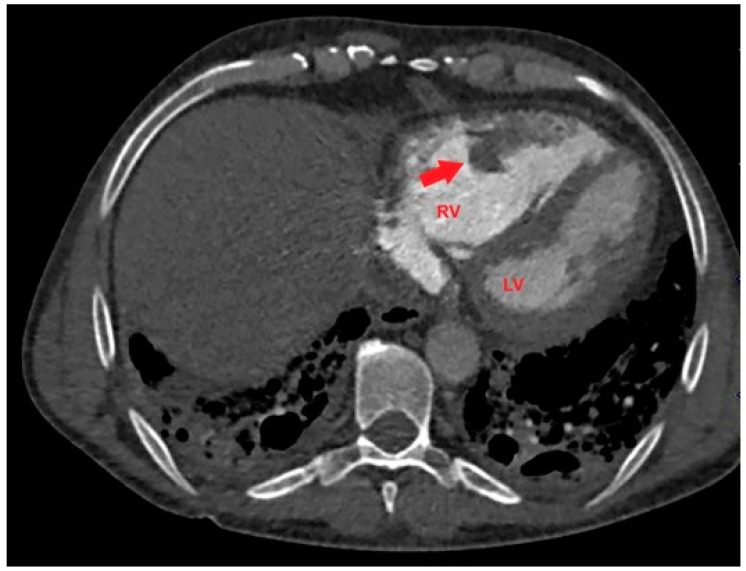
Axial contrast-enhanced chest computed tomography showing a large filling defect (red arrow) involving the mid-apical region of the right ventricle. LV, left ventricle; RV, right ventricle.

Based on the ECG and echocardiographic findings and the patient’s complex history, the pulmonologist suggested a diagnostic study with a contrast-enhanced chest CT. The examination confirmed severe RV dilatation with a large filling defect involving the mid-apical region of the right ventricle (Figure 4).

A CT scan also confirmed severe interstitial pulmonary fibrosis with diffuse ground glass opacities and thin-walled cysts and excluded PE.

A venous Doppler ultrasound of the lower extremities excluded concomitant DVT.

The patient was admitted to the Intensive Care Unit (ICU) and underwent high-flow oxygen therapy (reservoir mask with 10–15 L/min) and medical treatment with anticoagulants (enoxaparin sodium: 6000 I.U. twice daily by subcutaneous injection), IV antibiotics (Piperacillin/Tazobactam: 4 g/0.5 g three times daily), IV corticosteroids (methylprednisolone: 40 mg three times daily), IV antibiotics (ceftriaxone: 2 g daily) and IV diuretics (furosemide: 40 mg daily).

Even if the patient did not have a fever during hospitalization, aerobic and anaerobic blood cultures were performed to exclude the infectious origin of the RV mass. However, blood cultures yielded negative results.

**Figure 5:**
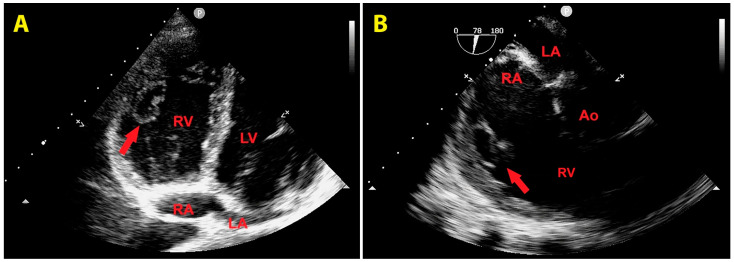
(**A**) Transthoracic echocardiography. Right-ventricular-focused apical four-chamber view demonstrating a slight reduction in right ventricular mass size (red arrow), characterized by an anechoic central space and a hyperechoic border. (**B**) Right-ventricular-focused mid-oesophageal section showing the same echocardiographic features of the right ventricular mass (red arrow), observed from the transthoracic approach. Ao, aorta; LA, left atrium; LV, left ventricle; RA, right atrium; RV, right ventricle.

A repeated TTE during the stay in the ICU demonstrated a slight reduction in RV mass size (3 cm × 2 cm) that changed its echogenicity, being characterized by an anechoic central space and a hyperechoic border (Figure 5A). The same echocardiographic findings of the RV mass were confirmed from an RV-focused mid-oesophageal section obtained during TEE (Figure 5B).

**Figure 6:**
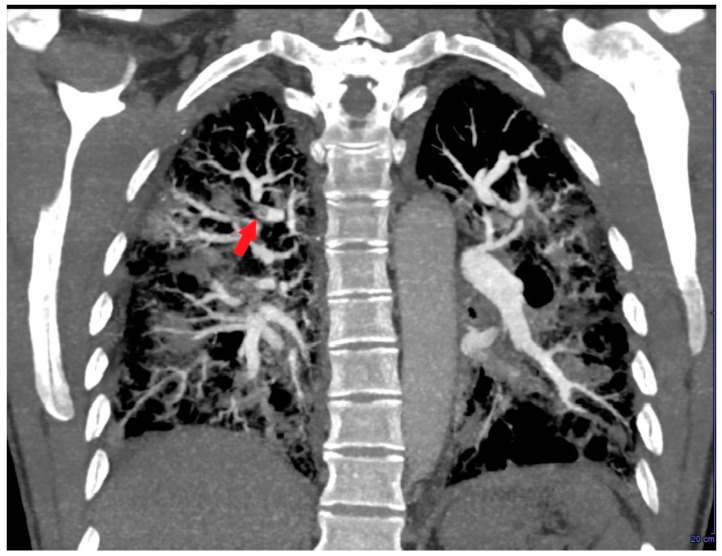
Coronal contrast-enhanced chest computed tomography revealing segmental and subsegmental filling defects (red arrow) in the right upper lobe.

A further contrast-enhanced chest CT scan revealed segmental and subsegmental filling defects in the right upper lobe (Figure 6) and a concomitant mild reduction in RV mid-apical filling defect.

**Figure 7:**
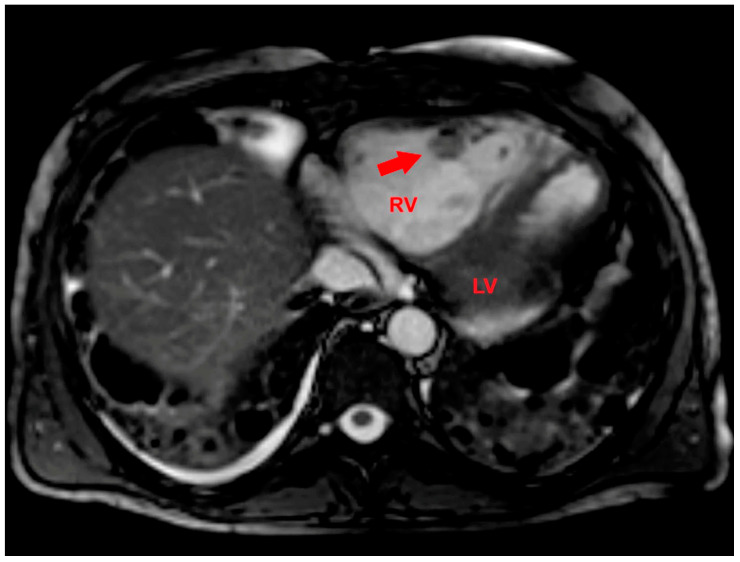
Axial late gadolinium enhancement cardiac magnetic resonance showing a right ventricular mass (red arrow) with inhomogeneous peripheral enhancement. LV, left ventricle; RV, right ventricle.

After a multidisciplinary discussion, late gadolinium enhancement (LGE) cardiac magnetic resonance (CMR) was performed. LGE-CMR allowed for the detection of an RV mass with inhomogeneous peripheral enhancement (Figure 7).

**Figure 8:**
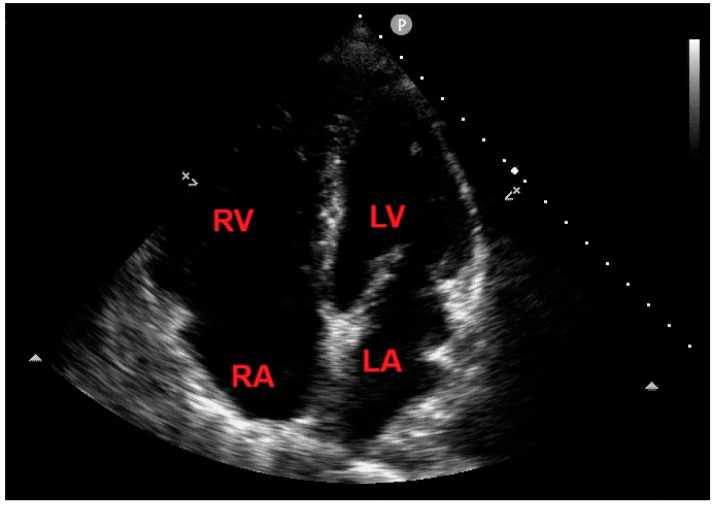
Transthoracic echocardiography. Apical four chamber view demonstrating the total disappearance of the right ventricular mass. LA, left atrium; LV, left ventricle; RA, right atrium; RV, right ventricle.

After seven days of ICU monitoring, the patient’s clinical conditions gradually improved, and he was transferred to the Division of Pneumology. On the 10th day of hospitalization, an echocardiographic control showed the total disappearance of the RV mass (Figure 8) and concomitantly improved TRV (estimated value: 2.5 m/s).

In light of the favourable echocardiographic evolution, the RV mass was more appropriately considered as a thrombotic formation with peripheral organization and liquefactive central necrosis, originating in situ, at the level of a significantly dilated and dysfunctional right ventricle, causing subsegmental PE, which was completely resolved after anti-coagulant treatment.

Repeated blood tests demonstrated the normalization of serum levels of CRP (2.8 mg/L) and troponin I (0.02 ng/mL) and a significant reduction in serum levels of both D-dimer (430 microg/L) and NT-proBNP (69 pg/mL). From a repeated blood gas analysis in ambient air, SaO2 was 91.4%, PaO2 was 59 mmHg, PaCO2 was 43 mmHg, pH was 7.41, and the lactate level was 1.01 mmol/L. During the hospital stay, the patient also underwent thrombophilia screening, which produced a negative result. The haematology consultant recommended anticoagulant therapy at discharge with 5 mg of apixaban twice daily lifelong.

On the 14th day of hospitalization, the patient was discharged with the diagnosis of acute-on-chronic respiratory failure secondary to acute exacerbation of interstitial lung disease, leading to acute RV overload with akinesia of the RV mid-apical segments, complicated by RV thrombosis in situ, causing subsegmental PE. The suggested discharge medical treatment included 5 mg of apixaban twice daily, oxygen therapy via the nasal cannula of 1 L/min at rest and 6 L/min on effort, 50 mg of azathioprine daily, and 25 mg of prednisone daily.

## 3. Discussion

This challenging clinical case highlights the complexity of the differential diagnosis of RV masses, involving tumours, vegetations, and thrombi.

### 3.1. Right Ventricular Tumors

RV neoplastic lesions may be benign or malignant. The most common benign cardiac tumours are myxomas, but those originating from the RV free wall are extremely rare, representing only 5% of cases [21,22]. RV myxomas may have obstructive features, potentially leading to right heart failure with systemic congestion or causing arrhythmias, syncope, and even sudden death [23]. Depending on their mobility, RV myxomas may be complicated by PE [24,25]. Rhabdomyomas represent another type of benign RV neoplastic lesion, predominantly detected in children and commonly associated with tuberous sclerosis complex; they are multiple lesions, that generally regress spontaneously [26].

Primary malignant cardiac tumours are generally right atrial masses that rapidly infiltrate valves or chamber walls destroy the primary structure of hearts [27]. Other characteristics of malignant tumours are the wide point of attachment, a size of >5 cm, pericardial effusion, and extracardiac extension [28]. Among the malignant cardiac tumours, the most frequent ones are cardiac sarcomas [29], particularly angiosarcomas, representing 30% to 45% of sarcomas [30]. Lymphomas are rare, accounting for 1% to 2% of primary malignant cardiac tumours, involving the right atrium or the right ventricle. Lymphomas are more frequently non-Hodgkin type, associated with immunodeficiency syndromes [28].

Distinctive features of primary malignant cardiac tumours assessed by CMR are heterogeneous enhancement, tumour necrosis, and multiple foci of calcification [31]. Compared with benign tumours, malignant masses have generally non-left localization, have a greater diameter, are sessile, are polylobate, have an inhomogeneous appearance, are infiltrating, and are accompanied by pericardial effusion. As opposed to the avascular nature of thrombi, cardiac tumours have a vascular supply. At LGE, malignant masses are more frequently hyperintense compared with benign ones [32].

Due to its high spatial and temporal resolution, fast acquisition times, and multiplanar image reconstruction capabilities, cardiac CT with ECG gating represents a valid alternative to CMR in many patients, particularly in those with contraindications to CMR. CT may precisely assess lesion margins, may define the cardiovascular extent of the mass, and exclude concomitant obstructive coronary artery disease [33]. Additionally, when combined with 18 F-fluorodeoxyglucose (FDG) positron emission tomography (PET), cardiac CT is also useful for detecting primary malignant cardiac tumours and metastasis, which generally show a significantly higher glucose uptake than primary benign cardiac tumours [34].

### 3.2. Right Ventricular Endocarditis

Right-sided infective endocarditis (IE), accounting for 5% to 10% of all IE cases, are generally associated with intravenous drug use, intracardiac devices, and central venous catheters, primarily involving the tricuspid valve and rarely, the pulmonary valve [35,36]. On the other hand, isolated RV mural endocarditis is very rare and generally arises from the RV moderator band, where trabeculations could act as a facilitating location for infection [37,38,39]. RV mural endocarditis may be suspected in patients with prolonged fever and RV endocardial masses, particularly attached to the RV moderator band, even when blood cultures are persistently negative. Cases of RV mural endocarditis involving the RV free wall have been reported in individuals who regular used intravenous drugs. These cases have been ascribed to the coarse trabeculae of the RV acting as a nidus for infection, similar to the RV moderator band [40,41].

The differential diagnosis between right-sided vegetations and thrombi may be difficult, because both are generally masses without gadolinium contrast enhancement [42]. However, a number of CMR findings may be suggestive of IE, particularly, delayed enhancement involving the cardiovascular structures, indicating endothelial inflammation, irreversible myocardial damage or fibrosis [43,44], LGE of the endothelial lining, or a perivalvular abscess [45]. Multislice CT may contribute to the diagnosis of right-sided IE, by providing a high-resolution anatomical assessment of vegetations, valvular and peri-valvular lesions, and also extra-cardiac lesions [46,47].

### 3.3. Right Ventricular Thrombosis

Given its higher sensitivity and specificity over echocardiographic techniques, CMR may facilitate the differential diagnosis between intracardiac thrombi and tumours, as demonstrated in various case reports and case series [13,14,48]. On CMR, first-pass perfusion imaging, early gadolinium enhancement, and delayed gadolinium enhancement with a prolonged inversion time are commonly used to assess the thrombotic nature of cardiac masses [49]. RVT is generally visualized as a homogeneously dark mass with no contrast uptake, characteristics that are consistent with a homogeneous, avascular mass [50]. However, peripheral enhancement may be occasionally observed in chronic organic thrombotic formations due to fibrotic components [51].

Another imaging technique commonly employed for the diagnostic study of RV masses is a chest CT scan, where RVT generally appears as a hypodense mass within enhanced RV cavity [52].

In the present case, a multidisciplinary evaluation and a dynamic multimodality imaging approach allowed for the correct differential diagnosis between RVT and both RV tumours and vegetation. Notably, an RV neoplastic lesion was excluded due to the absence of pericardial effusion and extracardiac extension and the rapid response to the anticoagulant treatment. Moreover, the infectious or inflammatory origin of the RV mass was not considered as a plausible hypothesis for the absence of a septic syndrome, for the negativity of blood cultures, and for the RV mass location, without any relation with the RV coarse trabeculae and/or the moderator band, which are generally considered possible sites for infection.

In our findings, the serial echocardiographic monitoring performed during hospitalization appeared to be more effective than contrast-enhanced chest CT and CMR for clarifying the real nature of the RV mass. During the acute phase, the bedside TTE raised the suspicion of severe PH complicated by mid-apical RV akinesia and a superimposed RV mass. The implementation of conventional TTE with PW-TDI provided a precise measurement of the RV mass’s mobility and embolic potential. In the sub-acute phase, TTE showed a slight reduction in RV mass size and identified a central anechoic area, confirmed by TEE examination. The subsequent contrast-enhanced chest CT confirmed the diagnosis of segmental and subsegmental PE, indicating that the reduction in RV mass size and central echogenicity were likely related to its fragmentation with consequent pulmonary embolization. The increased RV mass peak Va, assessed by PW-TDI in the acute phase, predicted its subsequent embolization early. Finally, after 10 days of hospitalization, TTE demonstrated the total disappearance of the RV mass, thus confirming its thrombotic nature.

In the present case, the thrombotic nature of the RV mass might have been suspected by the echocardiographic evidence of the underlying RV mid-apical free wall akinesia (similar to what has been observed for left ventricular apical aneurysms complicated by thrombosis) and by its gradual regression after the initiation of anticoagulant treatment. In light of our findings, the RVT we detected was a type B RVT, originating in situ and associated with significant RV dilatation and dysfunction.

For improving RV mass detection, we used colour PW-TDI rather than contrast echocardiography. As has been previously demonstrated [53], colour TDI may improve the visualization of intracardiac masses characterized by a pattern of motion that is totally different from that of surrounding myocardial structures. Indeed, these intracardiac masses are codified with different colours compared to the adjacent myocardium. Additionally, as was recently demonstrated by our study group [54], PW-TDI sampling of the free mobile portion of intracardiac pathological masses allows for the identification of their typical pattern of incoherent motion (totally asynchronous with respect to the cardiac walls) and the precise measurement of the mass peak Va. The higher the mass peak Va, the higher the risk of embolic complications [54]. In the present case, the patient was diagnosed with a mass peak Va >10 cm/s, thus confirming that this simple PW-TDI-derived parameter may represent an innovative important predictor of the embolic risk associated with mobile intracardiac masses.

### 3.4. Implications for Clinical Practice

PW-TDI is commonly employed for assessing the left ventricular filling pressures and/or the longitudinal systolic function of both ventricles. For this reason, the sample volume of PW-TDI is placed in the ventricular myocardium immediately adjacent to the mitral and/or tricuspid annulus [55]. Moreover, PW-TDI may be used for distinguishing between right atrial masses, characterized by uncoordinated motion, and pseudomasses, characterized by concordant motion with the surrounding myocardial tissue [56].

The assessment of cardiac masses’ mobility by PW-TDI is an innovative application of this imaging modality. To measure the mass peak Va, the sample volume of PW-TDI should be placed on the free mobile portion of the intracardiac mass. Even if it has not still been validated by multicentric studies, a mass peak Va > 10 cm/s may represent an adjunctive marker of the increased risk of systemic or pulmonary embolization, depending on the cardiac mass location. Accordingly, by analogy with what was demonstrated in patients with left ventricular apical thrombi [54], the echocardiographic detection of an RV mass peak Va > 10 cm/s may represent an important marker of increased embolic risk, thus strengthening the indication for prompt anticoagulant treatment.

## 4. Conclusions

The differential diagnosis of RV masses is complex, involving thrombi, tumours, and vegetations.

RVT should always be suspected in young patients with dilated and dysfunctional right ventricles and with systemic disorders associated with hypercoagulability.

A dynamic multimodality imaging approach, comprehensively incorporating colour and PW-TDI, may improve the visualization of RV masses, allowing clinicians to identify, among RV masses, those with an increased embolic potential.

## Data Availability

Data extracted from the present case report are publicly available on Zenodo (https://zenodo.org) (accessed on 15 February 2025).
